# Percutaneous Coronary Intervention Strategy for Spontaneous Coronary Artery Dissection of Left Main Coronary Artery with Extensive Intramural Hematoma in the Main Side Branch

**DOI:** 10.1155/2022/9679001

**Published:** 2022-02-15

**Authors:** Makio Muraishi, Kosuke Maeda, Takuya Okada, Masahiko Noguchi

**Affiliations:** ^1^Department of Cardiology, Tokyo Bay Urayasu Ichikawa Medical Center, Urayasu, Japan; ^2^Department of Cardiology, Yokosuka General Hospital Uwamachi, Yokosuka, Japan

## Abstract

A 46-year-old pregnant woman, presented with worsening episodes of intermittent chest pain. The patient was diagnosed with a non-ST-elevation myocardial infarction. On arrival, she had a stable hemodynamic status without chest pain. She was initially treated with conservative medical therapy. One day later, she complained of severe chest pain, and an electrocardiogram showed ST elevation in leads I, aVL, and V2-5. Emergency coronary angiography showed total occlusion of the left anterior descending artery (LAD) and intermediate stenosis of the left main coronary artery (LMCA). The intravascular ultrasound (IVUS) revealed an intramural hematoma (IMH) from the LMCA to the LAD, extending to the left circumflex artery (LCX) ostium. This finding was consistent with spontaneous coronary artery dissection (SCAD). After stent implantation from the LMCA to the LAD, severe stenosis was noted at the proximal site of the LCX. IVUS showed that the IMH extended to the LCX. The provisional crush stent technique was performed, and the final angiography revealed satisfactory results with thrombolysis in myocardial infarction flow grade 3 in the LAD and LCX. This case report highlighted that stent implantation in the SCAD lesions facilitated the extension of the IMH longitudinally and laterally into the side branch, resulting in stenosis or occlusion. Therefore, the side branch should be evaluated using IVUS before stent implantation. In cases where the IMH extends to the ostium of the side branch, two-stent techniques that do not require guidewire recrossing, such as crush stents, should be considered to avoid side branch occlusion.

## 1. Introduction

Spontaneous coronary artery dissection (SCAD) is defined as epicardial coronary artery dissection. It has been recognized as a significant cause of myocardial infarction, especially in younger women [1, 2]. Observational data have reported spontaneous angiographic healing in most patients (70-97%) [3, 4]. Meanwhile, percutaneous coronary intervention (PCI) for SCAD has been associated with high technical failure (36-53%) [3, 5]. In this procedure, the coronary guidewire may enter the false lumen and occlude the true lumen. Alternatively, balloon dilation and stent placement can result in extended intimal dissection, leading to intramural hematoma (IMH) formation, which propagates upstream and downstream. Thus, PCI for SCAD was recommended for patients, who have ongoing ischemia, cardiogenic shock, sustained ventricular arrhythmias, or left main coronary artery (LMCA) dissection, with a favorable anatomy for PCI [1]. However, the PCI strategy for SCAD has not been established. In this study, we describe a case of a 46-year-old woman, who presented with ST-elevation myocardial infarction (STEMI), caused by SCAD in the LMCA, treated with PCI. Furthermore, we discuss SCAD, including LMCA, focusing on the PCI strategy.

## 2. Case Series

A 46-year-old, five-week pregnant woman with hypertension and hyperlipidemia was transferred to our hospital with worsening episodes of intermittent chest pain. She experienced non-ST-elevation myocardial infarction (NSTEMI) due to type 2 SCAD, treated conservatively two years ago. Subsequently, she was maintained on antiplatelet therapy (aspirin 100 mg daily) and a beta blocker (bisoprolol 2.5 mg daily). On arrival, her chest pain disappeared, and she had stable vital signs. The physical examination was unremarkable, with no noted murmur, rub, gallop, or signs of cardiac enlargement. The initial electrocardiogram showed T-wave inversion in leads I, aVL, and V2-V4, but ST elevation or Q wave was not observed. Laboratory testing showed elevated cardiac enzymes, such as positive troponin T, creatinine phosphokinase (CK) (646 U/L), and CK-MB (54 U/L). Bedside transthoracic echocardiography revealed impaired contractility in the anterior and septal segments of the left ventricle. Since she was pregnant and hemodynamically stable without chest pain, she was treated conservatively with aspirin, nitroglycerin, heparin, and bisoprolol.

**Figure 1:**
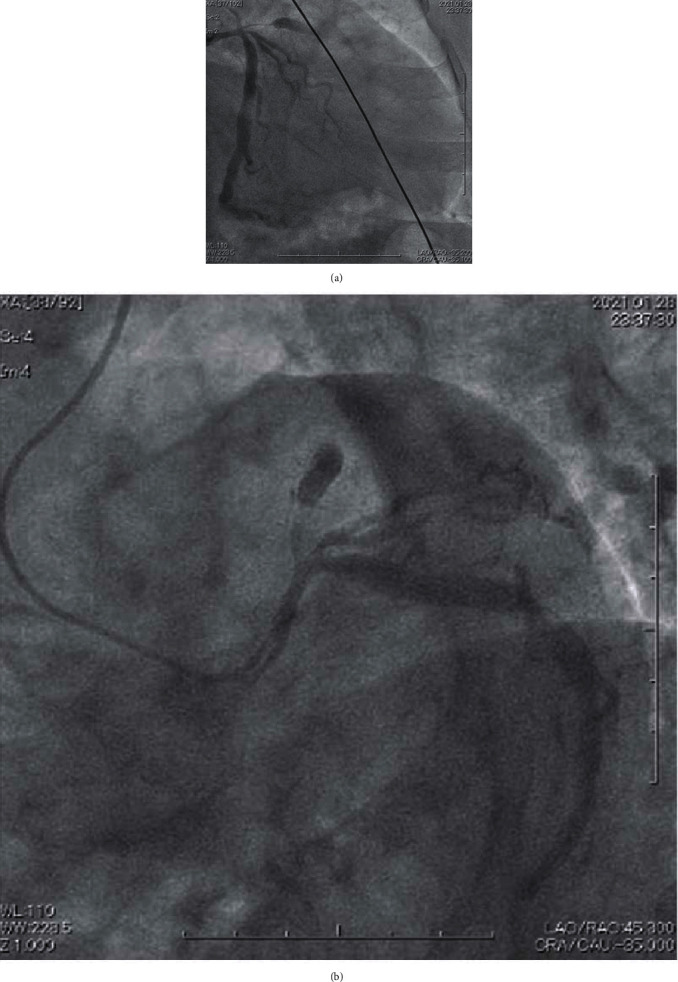
Emergency coronary angiography. (a) Right anterior oblique caudal projection and (b) left anterior oblique caudal projection showing TIMI flow 0 in the left anterior descending artery. Significant stenosis in the left main coronary artery and intermediate stenosis in the left circumflex artery were observed. In this case, the patient's abdomen and back were protected because she was pregnant, and the working view was limited to the right anterior oblique caudal projection and left anterior oblique caudal projection. TIMI: thrombolysis in myocardial infarction.

**Figure 2:**
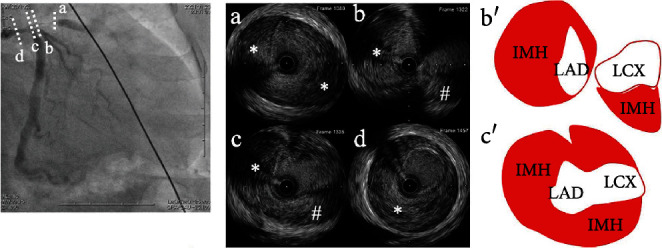
IVUS images of the left main coronary artery to left anterior descending artery and scheme of IVUS images. (a) IVUS image of the left anterior descending artery showing intramural hematoma (asterisk) compressing the true lumen. (b, b′) IVUS images of left anterior descending artery ostium showing intramural hematoma (asterisk). The left circumflex artery and intramural hematoma (sharp) extending to the left circumflex artery can also be observed. (c, c′) IVUS image of left main coronary artery bifurcation showing intramural hematoma extension to the left anterior descending artery (asterisk) and left circumflex artery (sharp). (d) IVUS image of left main coronary artery showing intramural hematoma (asterisk) compressing the true lumen. IVUS: intravascular ultrasound; LAD: left anterior descending artery; LCX: left circumflex artery; IMH: intramural hematoma.

**Figure 3:**
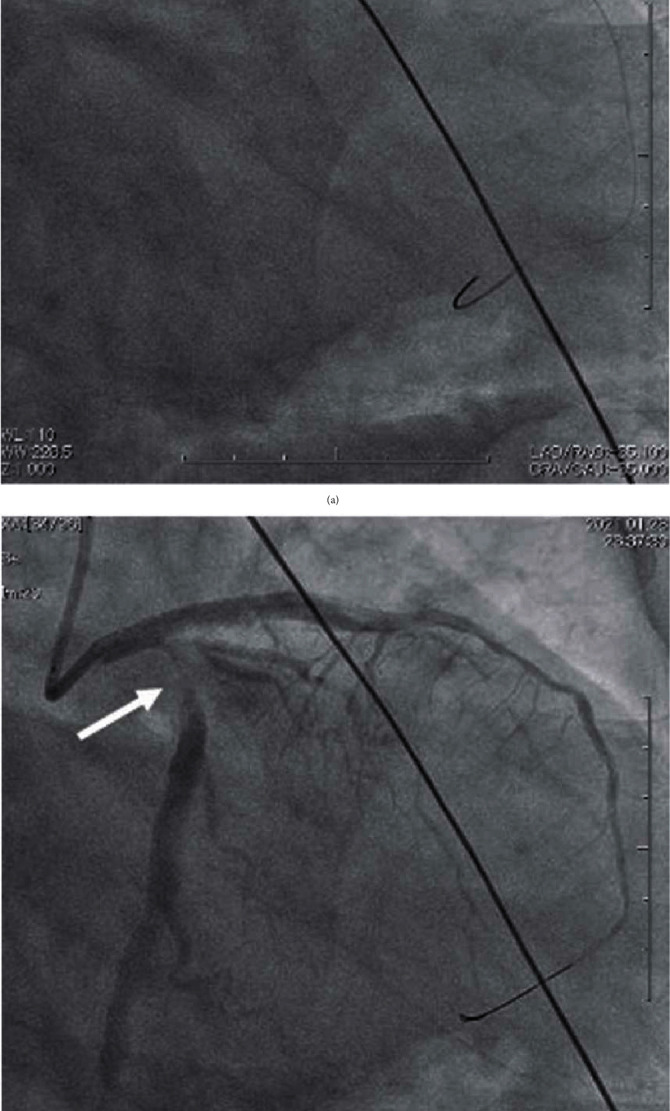
(a) The angiogram showing stent implantation from the left main coronary artery to the left anterior descending artery. (b) The angiogram after stent implantation. Significant stenosis appeared at the proximal site of the left circumflex artery (white arrow) which was not observed before stent implantation.

**Figure 4:**
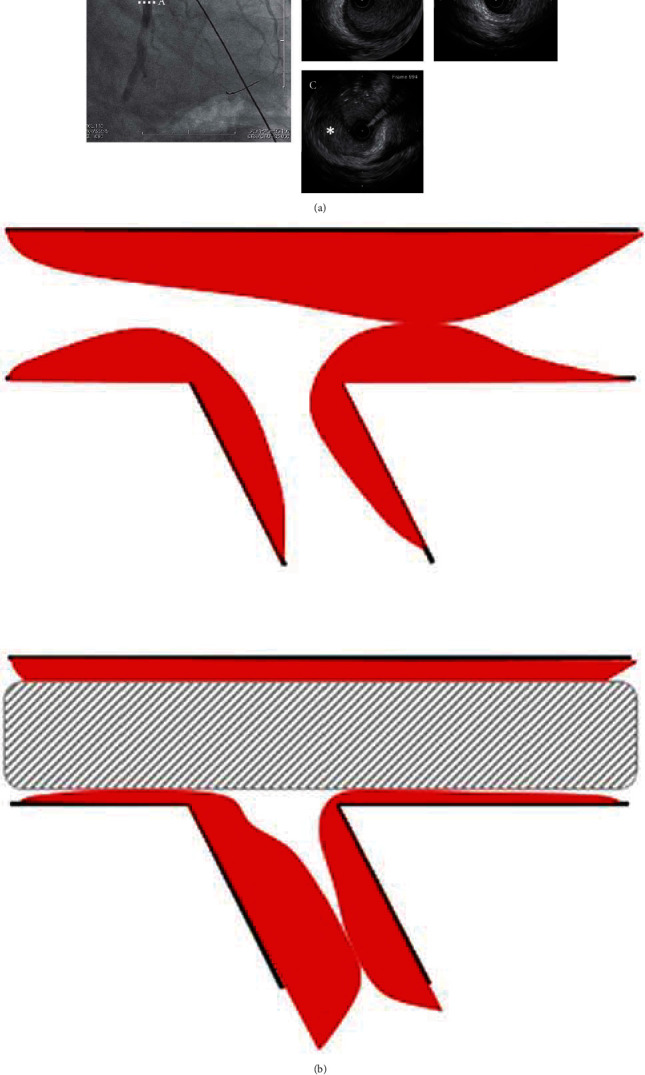
(a) The angiogram and IVUS images after stent implantation from the left main coronary artery to left anterior descending artery. (A) IVUS image of the midsite of left circumflex artery showing health vessel at this site. (B, C) IVUS image of the proximal site of left circumflex with significant stenosis showing huge intramural hematoma compressing the true lumen. (b) The scheme of extension of intramural hematoma to left circumflex after stent implantation from the left main coronary artery to left anterior descending artery.

**Figure 5:**
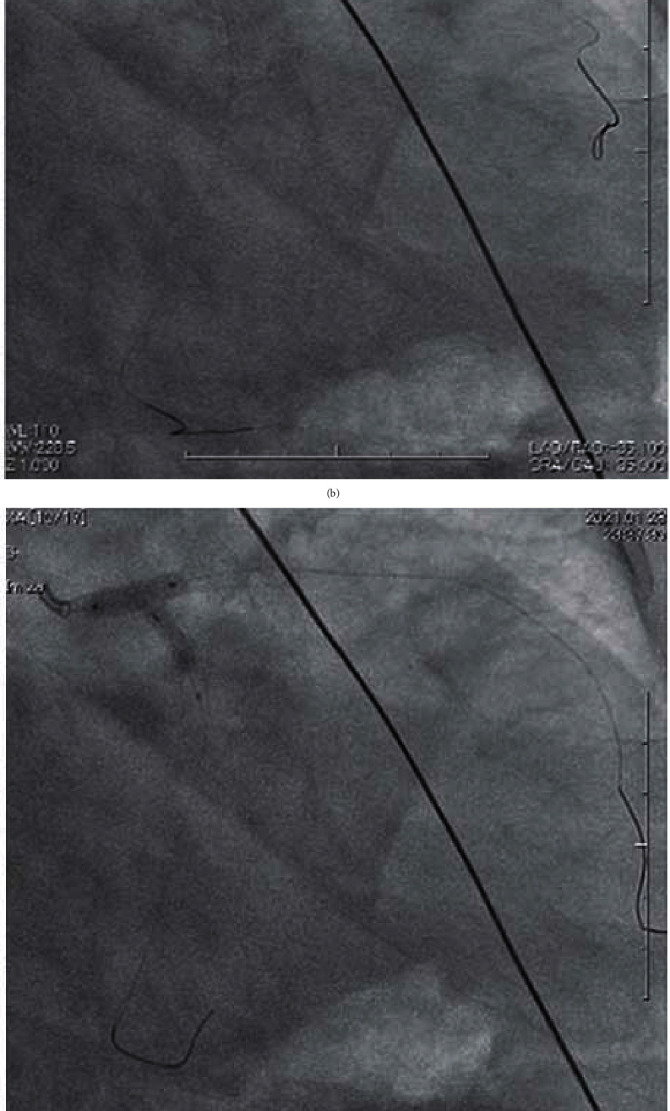
(a) The angiogram showing stent implantation from the left main coronary artery to the left circumflex artery through stent strut of the first stent. (b) The angiogram shows an inflated balloon at the left main coronary artery and a crushed stent. (c) The angiogram showing the kissing balloon technique after guidewire recrossing. (d) The final angiogram shows TIMI grade 3 flow in the left anterior descending artery and left circumflex artery.

One day later, she complained of severe chest pain, and an electrocardiogram showed ST elevation in leads I, aVL, and V2-5. Emergency coronary angiography revealed total occlusion of the left anterior descending artery (LAD) with thrombolysis in myocardial infarction (TIMI) flow grade 0 and intermediate stenosis of the LMCA (Figure 1). After the guidewire had been advanced into the LAD, the occluded lesion was dilated using a 2.0 mm coronary balloon, and the cause of the occlusion was determined via intravascular ultrasound (IVUS). IVUS revealed an intramural hematoma (IMH) from the LMCA to the LAD. The IMH extended to the left circumflex artery (LCX) ostium (Figure 2). Then, a 4.0 mm drug-eluting stent was implanted from the LMCA to the LAD to cover all IMHs. After stent implantation, a TIMI flow grade 3 was obtained in the LAD. However, the stenosis at the proximal segment of the left circumflex artery (LCX) progressed severely, and the LCX blood flow worsened to TIMI flow grade 2 (Figure 3). The guidewire was rewired through the stent struts. IVUS for LCX revealed a large IMH compressing the true lumen (Figure 4). Then, an additional drug-eluting stent (4.0 mm) was implanted into the LCX, using the provisional crush stent technique. The final angiography yielded satisfactory results with a TIMI grade 3 flow in both the LAD and LCX (Figure 5).

The postprocedural course was uneventful, and no major adverse cardiovascular events were observed. ECG before discharge revealed ST-segment resolution in the anterolateral leads. The patient's clinical course was uneventful during follow-up at the outpatient clinic.

## 3. Discussion

This case report highlighted that IMH can extend to the side branch. The PCI strategy for SCAD, including bifurcating lesions, should be carefully considered to avoid side branch occlusion. PCI has achieved a low success rate and high complication rate for SCAD due to difficulties in guidewire manipulation and the development of dissection and IMH [3, 5]. These challenging aspects are crucial, especially in bifurcation lesions, such as LMCA bifurcation. The present case illustrated two important lessons regarding the PCI strategy for SCAD.

First, intracoronary imaging was important in establishing the correct diagnosis and successfully performing PCI. It was difficult to distinguish between the true and false lumens using coronary angiography alone [6]. However, intracoronary imaging identified intimal tear, false lumen, IMH, and intraluminal thrombi [7]. Moreover, it confirmed the passage of the guidewire through the distal true lumen. Detecting the partial passage of the guidewire through the false lumen, including the bifurcation, allows manipulation of the guidewire into the true lumen under IVUS guidance. Numasawa et al. reported an IVUS-guided rewiring technique, which confirmed the passage of the guidewire through the true lumen of the LMCA and the false lumen of the LAD from the bifurcation. The second wire was inserted into the true lumen using a double-lumen catheter, placed in the true lumen of the LCX [8]. Intracoronary imaging can also be used to evaluate IMH expansion. In this case, severe stenosis was observed in the LAD, and the IMH was expected to extend mainly from the LMCA to the LAD. IVUS documented the expansion of the IMH from the LMCA to the LAD, and the extension of the IMH to the LCX ostium. This finding was a significant predictor of IMH propagation into the LCX after stent implantation in the LAD.

**Figure 6:**
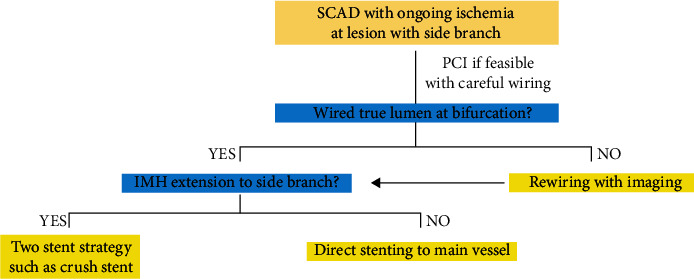
Proposed algorithm for management of SCAD with ongoing ischemia, which has the lesion crossing over the major side branch. SCAD: spontaneous coronary artery dissection; PCI: percutaneous coronary intervention; IMH: intramural hematoma.

Second, the one-stent strategy should be avoided in cases wherein the IMH of the main vessel extends into the side branch, because stent implantation in the main vessel can result in side branch occlusion secondary to IMH propagation, as seen in this case. When managing PCI for true LMCA bifurcation lesions due to atherosclerotic coronary artery disease, the stepwise provisional strategy can serve as the default intervention for distal LMCA bifurcation, rather than the two-stent strategy [9, 10]. However, for true LMCA bifurcation lesions due to SCAD, the provisional strategy harbors the risk of side branch occlusion due to IMH extension after stent implantation. Side branch occlusion secondary to IMH extension hinders the recrossing of another guidewire into the true lumen of the side branch through stent struts. In this situation, a two-stent strategy, such as a crush stent, is preferred because this technique does not require guidewire recrossing. This case, which involved a lesion crossing over the major side branch, documented the proposed PCI algorithm for SCAD (Figure 6).

SCAD is a common cause of myocardial infarction in pregnant or postpartum women [2]. Therefore, postoperative antiplatelet therapy is crucial when PCI is performed for SCAD in pregnant patients. Although there are few data on dual antiplatelet therapy (DAPT) during pregnancy, the European Society of Cardiology guideline states that no complications have been reported in stented pregnant patients treated with aspirin and clopidogrel and that the duration of DAPT can be shortened with second-/third-generation drug-eluting stents [11]. In this patient, DAPT (aspirin and clopidogrel) was introduced at the time of PCI since she was undergoing complex stenting of an LM bifurcation lesion. The P2Y12 inhibitor was planned for discontinuation before delivery with closed monitoring.

## 4. Conclusion

SCAD is an uncommon but important cause of acute coronary syndrome. Emergent PCI for SCAD remains challenging because of the difficulty of manipulating the guidewire into the true lumen and extensive IMH. When performing PCI for SCAD with an IMH, crossing over the major side branch, the extension of the IMH to the side branch should be evaluated using intracoronary imaging. In cases wherein the IMH extends to the side branch, a two-stent strategy that does not require guidewire recrossing should be considered to avoid side branch occlusion.

## Data Availability

The data supporting this case report are from previously studies and datasets, which have been cited.
